# Maximum isometric tongue force in patients with obstructive sleep apnoea

**DOI:** 10.1007/s00405-020-06327-7

**Published:** 2020-10-27

**Authors:** Richard Birk, Boris A. Stuck, Joachim T. Maurer, Angela Schell, C. Emika Müller, Benedikt Kramer, Stephan Hoch, J. Ulrich Sommer

**Affiliations:** 1 Department of Otorhinolaryngology Head and Neck Surgery, University Hospital Marburg, Philipps Universität Marburg, Baldingerstraße, 35039 Marburg, Germany; 2 Department of Otorhinolaryngology Head and Neck Surgery, University Hospital Mannheim, Mannheim, Germany; 3 Department of Otorhinolaryngology Head and Neck Surgery, University Hospital Wuppertal, Wuppertal, Germany

**Keywords:** Tongue, Force, Strength, OSA, Advancement, Suspension, Retention, Stabilization

## Abstract

**Background:**

Obstructive sleep apnea (OSA) is a sleep disorder with a prevalence of 9–38%. The underlying pathology in OSA is a collapse of the upper airway. Especially in more severely affected patients, this collapse is often located at the level of the tongue base. Therefore, various implantable systems (anchors and ligament techniques) were developed to prevent or overcome this collapse. These systems are exposed to various forces. Different models have been developed to measure these forces and data comparing forces in healthy individuals with OSA patients are rare.

**Purpose:**

Purpose of the study was to evaluate possible differences in tongue forces between healthy individuals and patients with OSA.

**Method:**

To evaluate maximum isometric tongue forces, we conducted a matched pair design study including 20 healthy individuals and 20 patients suffering from OSA. Maximum isometric tongue forces were measured in an anterior/posterior direction with the help of self-designed new device that clamps the tongue.

**Results:**

We could show that the maximum isometric force does not differ significantly in healthy individuals (10.7 ± 5.2N) from patients with OSA (14.4 ± 6.3N).

**Conclusion:**

Currently there are no indications that maximum isometric tongue force does differ in healthy individuals and patients with OSA. Higher, as well as lower, tongue forces in patients with OSA seem not to differ from healthy subjects and therefore may not be needed to consider, in the development of tongue management devices, for OSA patients.

## Introduction

The human tongue has to manage a couple of tasks necessary to live. This includes mastication, speech and swallowing. For the swallowing phase, in particular, the oral preparation, the oral transit and the pharyngeal phase, specific different forces are required [62]. It is also known, that reduction of the muscle tonus naturally occurs during sleep. This may contribute or intensify an obstruction of the upper airway in obstructive sleep apnea (OSA). OSA is a sleep disorder with a prevalence of 9–38%. Prevalence is increasing in the last years and is higher in different subgroups (e.g. male sex, presence of adipositas) of the population having a higher risk to develop OSA [19, 43, 44, 50, 63]. OSA associated symptoms are daytime sleepiness, non-restorative sleep and snoring [36, 45]. OSA is furthermore associated with an increased risk for cardiovascular morbidity and mortality [22, 29]. The underlying pathology in OSA is a collapse of the upper airway, leading to airway obstruction [10]. Although no difference of muscle activity (regarding the activation of motor units) for the strongest tongue muscle, the genioglossus muscle, when breathing in during wakefulness between healthy and OSA patients has been shown [30], in patients with OSA a collapses at the tongue base often occurs, especially in more severely affected patients[11, 59], even if the genioglossus muscle is higher activated compensatory at limited airflow during sleep [40].

The maximum isometric contraction forces of many human muscle groups has been well described in the literature [2]. However, literature regarding data of human tongue forces is rare. Only a few publications, evaluating the tongue strength during swallowing by measuring the swallowing pressure elicited by the anterior tongue, exist [37, 47, 61]. It was suggested that the maximum isometric force of the tongue may be higher than the tongue forces that occur during swallowing [62]. Nevertheless data about tongue mechanics, kinematics and the maximum tongue force is also rarely present. Two studies describe, that there is a gender dependency of maximum tongue strength in favor of men [8, 52]. They used the “Iowa Oral Performance Instrument” (IOPI). In these studies, the maximum elevation strength was assessed using a plastic bulb and pneumatic pressure sensor placed just behind the alveolar ridge. The maximum tongue force against the palate was estimated about 16 N [4, 23, 54]. It has also been shown that maximum tongue force seems to have a negative correlation with age in adults [37, 47, 52, 61]. But the participants of these studies were not healthy and consisted of patients with dysphagia or other swallowing disorders. Moreover most of the study groups who measured tongue strength have done this in a protruding way or the pressure was applied in a coronary direction [1, 12, 14, 34].

In the treatment of OSA, there are various tongue management systems available or under development to prevent retrolingual collapse. These systems include devices, implants or systems that retain, suspend, stabilize or advance the tongue. All these systems will be subject to a variety of load conditions and must consider forces, generated by the activation of the muscles in and around the tongue [16, 17, 58]. Probably the highest load on tongue management devices will be caused by activation of the styloglossus muscles. These muscles are activated during posterior movement of the tongue as for example during swallowing [17, 58]. The available tongue management devices include simple devices, moving the tongue forward by passive suction (AveoTSD®). Here, mixed results regarding OSA improvement are shown [20]. There are also implantable systems. One of these systems is a tongue advancing device incorporated into the tongue base, which can be adjusted postoperatively [17, 41, 58]. Again, the system was successful in some patients, but other patients experienced mechanical side effects or damages of the implanted system. These damages were probably due to the high mechanical stress caused by the tongue movements and forces [41, 58]. Tongue retention systems have also been brought up with different anchor, suture or ligament techniques. In these systems, a band is placed through the tongue base and fixed to the lower jaw [18, 31]. This type of tongue suspension system was first introduced in 1998 [7]. These systems must also withstand the maximum tongue force that may damage the retention system [18, 24].

Another treatment of OSA is the stimulation of the hypoglossal nerve with or without synchronization to the breathing cycle [9, 13, 53]. The muscle mainly responsible for airway stabilizing and opening during sleep is the above-mentioned genioglossus muscle, while other dilators do not seem to play a major role [38–40, 42, 49]. The devices themselves (especially the cables) do not have to withstand the full tongue force because they were largely implanted in a stress-free way, but, active and passive tongue forces must be overcome by the openers of the upper airway.

For that and the above-mentioned reasons (e.g. rare of data regarding healthy participants), we already investigated healthy participants in a tongue strength study. We were able to show that the maximum isometric tongue force in a posterior sagittal direction is 52.1 N and that there is a correlation with sex, body mass index (BMI) and tongue forces [51]. Very recently, Wirth et al. using the IOPI showed, that hypoglossal nerve stimulation therapy does not alter tongue protrusion strength and fatigability in obstructive sleep apnea [57].

However, it is still unclear if patients suffering from OSA compared to healthy individuals are able to achieve other maximum isometric tongue retaining forces and if tongue management devices therefore must be adopted to this situation. Therefore, the results of the present study might be relevant for the development of implants and other OSA management systems.

## Materials and methods

### Participants

The study was performed from May to November 2012 in a matched pairs design at the Sleep Disorders Center at the Department of Otorhinolaryngology, Head and Neck Surgery, Mannheim, Germany. Forty participants, 20 healthy subjects and 20 patients suffering from OSA were included. The participants were recruited before or after performing a diagnostic polysomnography (PSG). The study was approved by the local ethic committee (Medical Faculty Mannheim/University of Heidelberg). It was designed and performed in accordance to the Good Clinical Practice guidelines and the Declaration of Helsinki (EN ISO 14155). All participants agreed to the study protocol and written informed consent was obtained. Exclusion criteria included diseases of the musculoskeletal system, acute or chronic infections and major physical injury of the tongue in the participant’s past medical history. Additionally, we did not include patients with relevant abuse of additive substance. None of the study participants had an anatomical peculiarity (massive tonsil or tongue base hyperplasia, dislodging septal deviation etc.), which can be regarded as pathological.

### Measurement system and study protocol

**Fig. 1:**
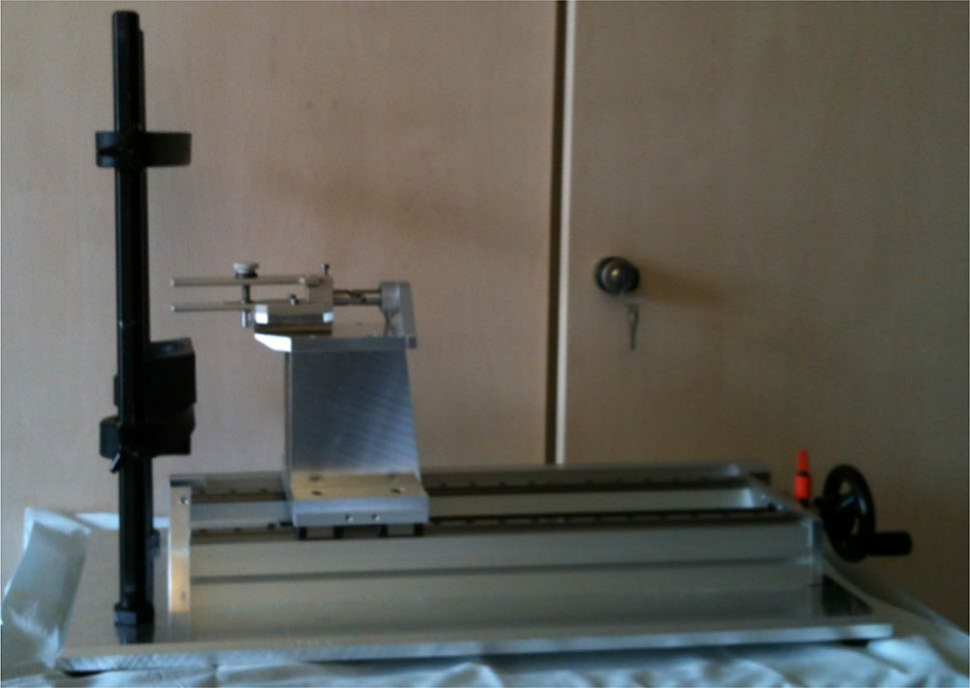
Picture of the measurement system

**Fig. 2:**
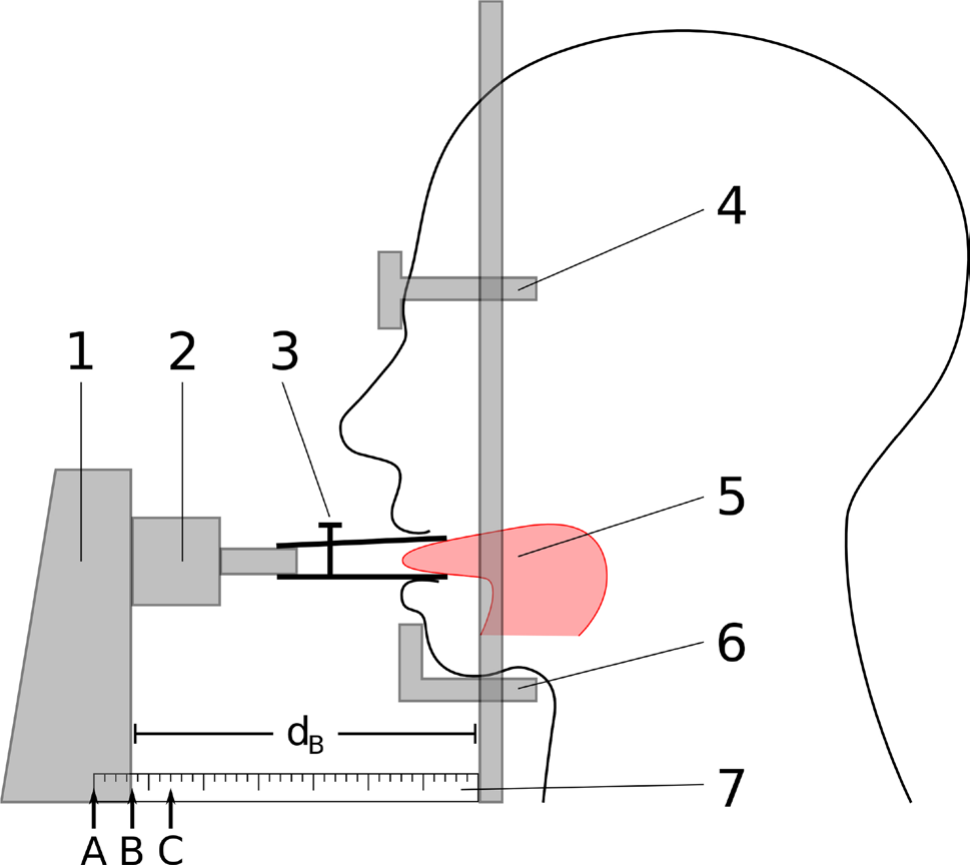
Tongue force measurement set-up in position** B** comprising of: **1** sliding mechanism, **2** force sensor, **3** tongue clamp, **4** headrest, **5** tongue, **6** chin rest, **7** ruler with the three different measurement positions (**A**, **B** and **C**) indicated. The distance of the sliding mechanism is depicted with dB [51]

As described in our first study, none of the previously discussed tongue force measurement systems proved to be useful for specific measurement of maximum retaining isometric tongue forces. To overcome this problem, a tongue fixating measuring system with a tongue clamp, consisting of two rigid plastic arms, in combination with a gauze compress surrounding the tongue used to fix the tongue, was developed [51]. This method is giving the firmest fixation of all methods tried [20]. Best results have been achieved, when the clamp and the compress were placed in the midline of the tongue in a way that ensures firm fixation of the tongue and in the same time not being to uncomfortable for the subject. The measurement system development was based on the work of Trawitzki et al. [54] and Kajee et al. [23]. The measurement system was designed in cooperation with and built by Philips Research Europe, and consists of a head positioning system (University of Houston/Houston/TX/USA), a tongue clamp, a piezo electric force sensor (Kistler Instrument Corp./Amherst, NY, USA) and a data logger (Centor Dual, two-channel force gauge, Andilog Technologies/Vitrolles/France). The schematic design and a picture of the system is shown in Figs. 1 and 2 [51].

In addition, age, gender, body height and body weight (from which body mass index (BMI) was calculated) were assessed for every subject and a sleep medical history was obtained. To assess the presence or absence of obstructive sleep apnea, all patients received a fully attended nocturnal PSG. The PSGs were conducted with hardware and software from Compumedics, Australia (Grael and Somté PSG headboxes with Profusion software version 3.0) and were evaluated according to the 2012 update of the 2007 procedures and definitions of the American Academy of Sleep Medicine (AASM) [3]. Hypopnea was a drop in peripheral oxygen saturation of 3% and a reduction in respiratory flow of between 30 and 90% for at least 10 s compared to baseline. OSA was defined as apnea hypopnea index ${\text{AHI}} \le 5$.

Short after, a theoretical introduction into the measurement system was given to all participants. After system adjustments and explanation not to use the neck muscles during the tests, the participants then were measured three times in all following tongue positions. Position A, “maximal tongue protrusion”, position B, a “neutral or resting” position and position C, a slightly “retracted” tongue position. To overcome the “learning effect”, we observed in our last study, the participants could try the measurement system within the introduction and adjustment. The exact study-protocol can be found in [51].

### Analysis

Statistical analysis and plotting was done using “R”, an open source environment for statistical computing and graphics [46].

For testing of the normal distribution, the Kolmogorov–Smirnov test was used. Since the data did not appear to be normally distributed and the groups are dependent of each other in specific test situations, the Wilcoxon signed-rank test as non-parametric statistical hypothesis test was used to measure statistical dependence between the variables. For the evaluation of correlations between tongue force, BMI, age and OSA, the Spearman test was used.

## Results

### Demographic parameters of participants

27 male and 13 female participants were included in the study. The mean age of all participants was 48.3 ± 14.11 years with a minimum age of 21 and a maximum age of 75 years. The mean of all participants BMI was 27.8 ± 3.6 kg/m^2^ ranging from 19 to 34.7 kg/m^2^. There was no significant difference regarding the age, BMI and the sex between the healthy and OSA group. Five women suffered from OSA and all were postmenopausal age ($$ \ge \ $$51 years). Eight women did not suffer from OSA and 4 of them were $$ \ge \ $$51 years old. Six women were aged from 45 to 55 years and 4 of them showed no OSA. Included subjects were non-smoker and non-alcohol user. The mean AHI in healthy patients was 1.9±1.7/h ranging from 0 to 4.9/h and the mean AHI in patients with OSA was 33.7±15.8/h ranging from 15 to 61/h. Details, separated into OSA and healthy participant, are shown in Table 1.

**Table 1:** Study participants details

	Healthy	OSA
*N*	20	20
Age (y)	46.6 ± 16.2	50.1 ± 12.3
Sex (m/f)	12/8	15/5
BMI	27.5 ± 3.9	28.2 ± 3.4
BMI male	28 ± 3.5	28.2 ± 3.3
BMI female	25.8 ± 3.5	28.2 ± 3.4
AHI	1.9 ± 1.6	33.7 ± 15.8
AHI male	2.5 ± 1.4	33.2 ± 14.4
AHI female	0.9 ± 1.6	36.6 ± 18.8

*N* number, *y* years, *m* male, *f* female, *BMI* body mass index (kg/m^2^), *AHI* apnea/hypopnea index

### Tongue strength and OSA

The mean maximum force in all male participants was 13.6±6N ranging from 5.3 to 31.8N. Mean maximum force in all female participants was 10.5±5.8N ranging from 5 to 23.3N. Male participants showed higher forces than female participants (*p* < 0.001).

Looking at all participants, we could measure significant lower forces in older participants (rho = − 0.2499, *p* < 0.001).

**Fig. 3:**
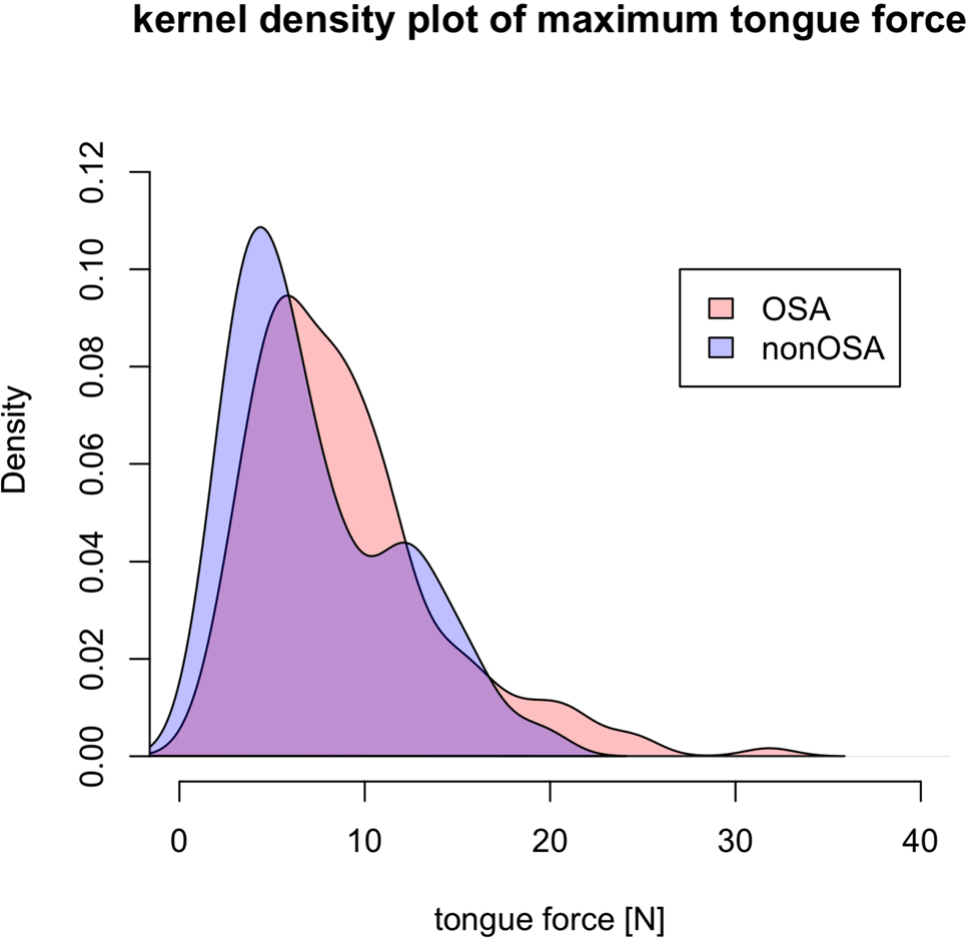
Kernel density plot of the maximum force comparing healthy participants and participants with obstructive sleep apnea (OSA)

The OSA-group and the healthy group did not differ significantly in sex, age, BMI and evaluated maximum forces. Table 2 shows force details and Fig. 3 gives graphical interpretation. Even after the healthy- and OSA-participants were divided into men and women, there were no significant differences between the groups.

**Table 2:** Max. force details for healthy participants and participants with OSA. Max. force in Newton

	Healthy	OSA
All		
$\varnothing$	10.7±5.2	14.4±6.3
Min/Max	3.5/19.8	5.4/31.8
Male		
$\varnothing$	11.6±4.8	15.1±6.3
Min/Max	5.3/19.9	5.4N/31.8
Female		
$\varnothing$	9.3±5.5	12.4±5.7
Min/Max	5/19.8	7.4/23.3

## Discussion

Various tongue management systems were developed for the treatment of patients with OSA. Until now, the maximum forces in patients with OSA compared to healthy individuals in the sagittal retaining direction and thus the forces effecting the tongue including devices are still unclear. This study describes differences in maximum isometric forces in healthy patients compared to patients with OSA in an isolated anterior–posterior direction. These maximum forces were systematically evaluated in 40 participants, 20 of them healthy and 20 of them suffering from OSA in a matched pair design study. The measurements were collected three times in each individual of three different tongue positions, as we described in a previous study, where we evaluated the maximum isometric tongue forces in 59 healthy individuals and showed it is 52.1 N in an anterior–posterior direction [51]. After trying several mechanisms of fixating, the tongue in a sagittal direction (e.g., suction cups or adhesive strips), the tongue clamp depicted in Fig. 1 was developed, but the clamp is just a compromise between rigid fixation and participant comfort when applying high tongue tension [51]. As with our previous study main problem was the pain caused by the clamp. Very recently Wirth et al showed, that hypoglossal nerve stimulation therapy does not alter tongue protrusion strength and fatigability in obstructive sleep apnea. Tongue strength measurement was performed using the IOPI with bulb placement behind the alveolar ridge/hard palate. They also investigated tongue strength differences in healthy participants. After multiple linear regressions analysis, they could show, that only age was a significant variable contrary to BMI or presence of OSA [57].

### Gender, BMI and age

There is a significant gender difference with higher forces in male participants. This is in line with our findings in our last study [51]. The higher maximum value in male participants could also be shown in other studies using different methodologies and analyzing different force directions [28, 32, 54, 61]. Interestingly, there seems to be no significant difference in fatigability indices between men and women [33]. Surprisingly, other studies showed that the medium tongue strength in swallowing and swallowing pressure displayed no gender difference [6, 56, 60, 62]. An explanation could be, that other measuring systems were used in this studies (e.g. the IOPI or the “Kay Swallowing work station” where pneumatic pressure sensors are placed at the palate) and the measured force direction is different.

The BMI did not correlate with the maximum achievable isometric tongue force, regardless if the groups were split into OSA and healthy or not. This is in contrast to our study in healthy participant, where we could show a positive correlation between BMI and maximum isometric tongue force, but is in line with the most recent study of Wirth et al. [57]. The present data agrees with a study of Mortimore et al. in 1999. He concluded that lingual protrusion fatigability was not correlated with the variables of fat-free mass, weight or height [33]. In another study by Carrera et al., in vitro genioglossus endurance was higher in non-obese OSA patients but not in obese OSA patients compared to controls. They could not show difference in genioglossus endurance in CPAP-treated non-obese OSA patients [5]. The comparability to our study is limited since fatigue was tested in vitro after a genioglossus biopsy. In another study, lower tongue total muscle work was detected in non-obese compared to untreated obese patients with moderate to severe OSA [25]. Summarizing the studies BMI seems not to be influencing tongue fatigue in treated non-obese OSA patients but in untreated non-obese OSA patients.

The literature is lacking data regarding isometric tongue force but a study by Vaara et al. showed that total body mass and fat correlates negatively with muscular endurance, while the fat free mass and maximal isometric strength correlates positively [55]. Fat-free mass and BMI have a positive correlation in most non-obese populations. It was suggested that the maximum tongue force is increased in people with an higher BMI, due to increased tongue exercise from increased food intake [24, 51]. We could not confirm the expectation that there is a positive correlation between BMI and maximum isometric tongue force (especially because there is a positive correlation between BMI and obstructive sleep apnea [15]) as in our last study. In conclusion, the available data shows controversial results, even to our previous study.

Age and maximum tongue force correlate statistically significant in a negative manner. Younger participants achieved higher maximum isometric forces. This confirms the data of our last study [51] and is in line with the above mentioned work of Mortimore et al. and may be related to the loss of muscle mass during the aging process [33]. Also Wirth et al. showed that there was a significant correlation between tongue strength and age [57]. Other studies also displayed the loss of maximum tongue strength in older individuals. The strength loss in elderly is also known for other muscles like the biceps brachii [2, 7, 32, 37, 47, 62].

Besides that, the influence of estrogen on muscle contractility must be considered. In a rat model, it was demonstrated that the contractility of genioglossus was accentuated by estrogen. Moreover, these effects were at least in part, meditated directly via regulation of the expression of estrogene receptor [21]. This estrogenic effects have been observed in natural (phyto-)estrogens and synthetic derivative and possible responsible proteins have been identified [26, 27, 64]. The findings might contribute to a protective effects of estrogen on the pathogenesis of oOSA. It is to be expected that these effects observed in the genioglossus also affect the other muscles of the tongue and thus the strength and endurance of the muscles changes in postmenopausal women. Actually, all included woman showing an OSA were postmenopausal, what might bias our results.

### Difference between OSA and healthy

No significant tongue force differences could be measured between healthy participants and participants suffering from OSA, no matter if the groups were divided into male and female. In this study, patients with OSA did not show significant stronger or lower force, so we could not confirm our initial expectation, that patient with OSA have stronger isometric tongue forces, although looking at the data shows a non-significant trend to stronger tongue forces in patients with OSA. This might have been important in constructing and developing tongue systems implanted in and around the tongue like tongue retaining systems. In addition, the system and the study design were developed, among other things, to measure the tongue retraction forces, since the anchors of the tongue anchor systems were often broken. Interestingly Wirth et al. could show that OSA had a significant influence on tongue endurance [57]. Nevertheless, a tongue force measurement system, implemented in clinical routine, could be useful in patients with OSA prior to implantation of tongue retaining systems as a predictor of success or even as marker for exclusion. Therefore, the measurement system could be an outcome marker to generate a predictive value but further studies are needed here, due to the fact that data regarding a possible correlation between the efficiency, durability and the rate of failure of tongue retaining systems and the maximum isometric tongue retracting force is missing up to date.

Finally, it has to be mentioned that there are still limitations of the study: there are several aspects to be discussed about the study design, mainly about the measurement system. The force measurement was only performed in an anterior–posterior way so posterior–anteriorly directed and other directed tongue forces might be underestimated and could not be measured or included in the results. The force during protrusion is certainly much more important for keeping the pharynx open than the force development during retraction, since the pharynx closes and does not open when the tongue is retracted. With regard to this and obstructive sleep apnea, the above-mentioned study by Wirth et al. showed that tongue strength in patients with OSA compared to healthy individuals had no significant differences in tongue strength but had lower tongue endurance [57]. Due to the maximum protruding measurement position intrinsic muscle fibres (transverse and vertical) are preloaded and, therefore, might be involved in the tongue retracting movement [35, 48]. Taking into account the publication just mentioned our measurement system probably mainly measures the activation of this intrinsic muscles. Yet, possibilities for testing the maximum isometric tongue force in a posterior sagittal direction are very limited and therefore have not been done. We finally came up with the above measurement setup as the best available regarding our question. It gives a rough estimation of the above-mentioned tongue force but reliability would probably have to be proved in further studies. To avoid mislead conclusion, the data of the study have to be handled with care. This study also cannot claim to measure in detail the force of the main opener of the airway (musculus genioglossus). However, this was not the primary intention of the authors, but this has to be involved in the interpretation of the data. Another limitation is the above-mentioned uncomfortable, maybe painful, clamp fixation of the tongue during the measurements and, therefore, the maximum measured forces could be reduced. However there is no other, more suitable, system available up to date to measure specific retaining forces [14, 31, 34, 41].

## Conclusion

Currently, there are no indications that maximum isometric tongue force does differ in healthy individuals and patients with OSA. Higher as well as lower tongue forces in patients with OSA might not need to be considered in the development of tongue management devices for OSA patients but the limitations of the study need to be considered.

## References

[1] Adams V, Mathisen B, Baines S, Lazarus C, Callister R (2013). A systematic review and meta-analysis of measurements of tongue and hand strength and endurance using the Iowa Oral Performance Instrument (IOPI). Dysphagia.

[2] Avin KG, Law LA (2011). Age-related differences in muscle fatigue vary by contraction type: a meta-analysis. Phys Ther.

[3] Berry RB, Budhiraja R, Gottlieb DJ, Gozal D, Iber C, Kapur VK, Marcus CL, Mehra R, Parthasarathy S, Quan SF (2012). Rules for scoring respiratory events in sleep: update of the 2007 AASM manual for the scoring of sleep and associated events. J Clin Sleep Med.

[4] Busha BF, Strobel RJ, England SJ (2002). The length-force relationship of the human genioglossus in patients with obstructive sleep apnea. Respir Physiol Neurobiol.

[5] Carrera M, Barbe F, Sauleda J, Tomas M, Gomez C, Santos C, Agusti A (2004). Effects of obesity upon genioglossus structure and function in obstructive sleep apnoea. Eur Respir J.

[6] Clark HM, Henson PA, Barber WD, Stierwalt JA, Sherrill M (2003). Relationships among subjective and objective measures of tongue strength and oral phase swallowing impairments. Am J Speech Lang Pathol.

[7] Coleman J, Bick PA (1999). Suspension sutures for the treatment of obstructive sleep apnea and snoring. Otolaryngol Clin North Am.

[8] Crow HC, Ship JA (1996). Tongue strength and endurance in different aged individuals. J Gerontol A Biol Sci Med Sci.

[9] Eastwood PR, Barnes M, MacKay SG, Wheatley JR, Hillman DR, Nguyên X-L, Lewis R, Campbell MC, Pételle B, Walsh JH (2020). Bilateral hypoglossal nerve stimulation for treatment of adult obstructive sleep apnoea. Eur Respir J.

[10] Edwards BA, Eckert DJ, Jordan AS (2017). Obstructive sleep apnoea pathogenesis from mild to severe: is it all the same?. Respirology.

[11] Eichler C, Sommer JU, Stuck BA, Hormann K, Maurer JT (2013). Does drug-induced sleep endoscopy change the treatment concept of patients with snoring and obstructive sleep apnea?. Sleep Breath.

[12] Freedman DS, Wang J, Maynard LM, Thornton JC, Mei Z, Pierson RN, Dietz WH, Horlick M (2005). Relation of BMI to fat and fat-free mass among children and adolescents. Int J Obes (Lond).

[13] Friedman M, Jacobowitz O, Hwang MS, Bergler W, Fietze I, Rombaux P, Mwenge GB, Yalamanchali S, Campana J, Maurer JT (2016). Targeted hypoglossal nerve stimulation for the treatment of obstructive sleep apnea: six-month results. Laryngoscope.

[14] Furlan RM, Motta AR, Valentim AF, Barroso MF, Costa CG, Casas EB (2013). Protrusive tongue strength in people with severely weak tongues. Int J Speech Lang Pathol.

[15] Gordon AM, Huxley AF, Julian FJ (1966). The variation in isometric tension with sarcomere length in vertebrate muscle fibres. J Physiol.

[16] Hamans E, Boudewyns A, Stuck BA, Baisch A, Willemen M, Verbraecken J, Heyning P, Van D (2008). Adjustable tongue advancement for obstructive sleep apnea: a pilot study. Ann Otol Rhinol Laryngol.

[17] Hamans E, Shih M, Roue C (2010). A novel tongue implant for tongue advancement for obstructive sleep apnea: feasibility, safety and histology in a canine model. J Musculoskelet Neuronal Interact.

[18] Handler E, Hamans E, Goldberg AN, Mickelson S (2014). Tongue suspension: an evidence-based review and comparison to hypopharyngeal surgery for OSA. Laryngoscope.

[19] Heinzer R, Vat S, Marques-Vidal P, Marti-Soler H, Andries D, Tobback N, Mooser V, Preisig M, Malhotra A, Waeber GUA (2015). Prevalence of sleep-disordered breathing in the general population: the HypnoLaus study. Lancet Respir Med.

[20] Higurashi N, Kikuchi M, Miyazaki S, Itasaka Y (2002). Effectiveness of a tongue-retaining device. Psychiatry Clin Neurosci.

[21] Hou YX, Jia SS, Liu YH (2010). 17$$\beta $$-Estradiol accentuates contractility of rat genioglossal muscle via regulation of estrogen receptor $$\alpha $$. Arch Oral Biol.

[22] Javaheri S, Barbe F, Campos-Rodriguez F, Dempsey JA, Khayat R, Javaheri S, Malhotra A, Martinez-Garcia MA, Mehra R, Pack AI (2017). Sleep apnea: types, mechanisms, and clinical cardiovascular consequences. J Am Coll Cardiol.

[23] Kajee Y, Pelteret JP, Reddy BD (2013). The biomechanics of the human tongue. Int J Numer Method Biomed Eng.

[24] Kezirian EJ, Goldberg AN (2006). Hypopharyngeal surgery in obstructive sleep apnea: an evidence-based medicine review. Arch Otolaryngol Head Neck Surg.

[25] Li W-Y, Gakwaya S, Saey D, Sériès F (2017). Assessment of tongue mechanical properties using different contraction tasks. J Appl Physiol.

[26] Li Y, Liu Y, Lu Y, Zhao B (2017). Inhibitory effects of 17$$\beta $$-estradiol or a resveratrol dimer on hypoxia-inducible factor-1$$\alpha $$ in genioglossus myoblasts: Involvement of ER$$\alpha $$ and its downstream p38 MAPK pathways. Int J Mol Med.

[27] Lu Y, Liu Y, Li Y (2014). Comparison of natural estrogens and synthetic derivative on genioglossus function and estrogen receptors expression in rats with chronic intermittent hypoxia. J Steroid Biochem Mol Biol.

[28] Lujan-Climent M, Martinez-Gomis J, Palau S, Ayuso-Montero R, Salsench J, Peraire M (2008). Influence of static and dynamic occlusal characteristics and muscle force on masticatory performance in dentate adults. Eur J Oral Sci.

[29] Lurie A (2011). Cardiovascular disorders associated with obstructive sleep apnea. Adv Cardiol.

[30] Luu BL, Saboisky JP, McBain RA, Trinder JA, White DP, Taylor JL, Gandevia SC, Butler JE (2019). Genioglossus motor unit activity in supine and upright postures in obstructive sleep apnea. Sleep.

[31] Miller FR, Watson D, Malis D (2002). Role of the tongue base suspension suture with the Repose System bone screw in the multilevel surgical management of obstructive sleep apnea. Otolaryngol Head Neck Surg.

[32] Mortimore IL, Bennett SP, Douglas NJ (2000). Tongue protrusion strength and fatiguability: relationship to apnoea/hypopnoea index and age. J Sleep Res.

[33] Mortimore IL, Fiddes P, Stephens S, Douglas NJ (1999). Tongue protrusion force and fatiguability in male and female subjects. Eur Respir J.

[34] Motta AR, Cesar CC, Bommarito S, Chiari BM (2011). Axial force of the tongue in different age groups. J Soc Bras Fonoaudiol.

[35] Mu L, Sanders I (2010). Human tongue neuroanatomy: nerve supply and motor endplates. Clin Anat.

[36] Newman AB, Nieto FJ, Guidry U, Lind BK, Redline S, Pickering TG, Quan SF, Sleep Heart Health Study Research G (2001). Relation of sleep-disordered breathing to cardiovascular disease risk factors: the Sleep Heart Health Study. Am J Epidemiol.

[37] Nicosia MA, Hind JA, Roecker EB, Carnes M, Doyle J, Dengel GA, Robbins J (2000). Age effects on the temporal evolution of isometric and swallowing pressure. J Gerontol A Biol Sci Med Sci.

[38] Oliven A, Odeh M, Schnall R (1996). Improved upper airway patency elicited by electrical stimulation of the hypoglossus nerves. Respiration.

[39] Oliven A (2011). Treating obstructive sleep apnea with hypoglossal nerve stimulation. Curr Opin Pulm Med.

[40] Oliven R, Cohen G, Somri M, Schwartz AR, Oliven A (2020). Relationship between the activity of the genioglossus, other peri-pharyngeal muscles and flow mechanics during wakefulness and sleep in patients with OSA and healthy subjects. Respir Physiol Neurobiol.

[41] Pavelec V, Hamans E, Stuck BA (2011). A study of the new generation of the advance system tongue implants: three- and six-month effects of tongue to mandible tethering for obstructive sleep apnea. Laryngoscope.

[42] Pengo MF, Steier J (2015). Emerging technology: electrical stimulation in obstructive sleep apnoea. J Thorac Dis.

[43] Peppard PE, Young T, Barnet JH, Palta M, Hagen EW, Hla KM (2013). Increased prevalence of sleep-disordered breathing in adults. Am J Epidemiol.

[44] Punjabi NM (2008). The epidemiology of adult obstructive sleep apnea. Proc Am Thorac Soc.

[45] Quan SF, Howard BV, Iber C, Kiley JP, Nieto FJ, O’Connor GT, Rapoport DM, Redline S, Robbins J, Samet JM, Wahl PW (1997). The Sleep Heart Health Study: design, rationale, and methods. Sleep.

[46] R Core Team (2020) R: A language and environment for statistical computing. R Foundation for Statistical Computing, Vienna, Austria. URL https://www.R-project.org/

[47] Robbins J, Levine R, Wood J, Roecker EB, Luschei E (1995). Age effects on lingual pressure generation as a risk factor for dysphagia. J Gerontol A Biol Sci Med Sci.

[48] Sanders I, Mu L (2013). A three-dimensional atlas of human tongue muscles. Anat Rec.

[49] Schwartz AR, Smith PL, Oliven A (2013). Electrical stimulation of the hypoglossal nerve: a potential therapy. J Appl Physiol.

[50] Senaratna CV, Perret JL, Lodge CJ, Lowe AJ, Campbell BE, Matheson MC, Hamilton GS, Dharmage SC (2017). Prevalence of obstructive sleep apnea in the general population: a systematic review. Sleep Med Rev.

[51] Sommer JU, Birk R, Hörmann K, Stuck BA (2014). Evaluation of the maximum isometric tongue force of healthy volunteers. Eur Arch Oto-Rhino-Laryngol.

[52] Stierwalt JA, Youmans SR (2007). Tongue measures in individuals with normal and impaired swallowing. Am J Speech Lang Pathol.

[53] Strollo PJ, Soose RJ, Maurer JT, Vries N, de Cornelius 
J, Froymovich O, Hanson RD, Padhya TA, Steward DL, Gillespie MB (2014). Upper-airway stimulation for obstructive sleep apnea. N Engl J Med.

[54] Trawitzki LV, Borges CG, Giglio LD, Silva JB (2011). Tongue strength of healthy young adults. J Oral Rehabil.

[55] Vaara JP, Kyrolainen H, Niemi J, Ohrankammen O, Hakkinen A, Kocay S, Hakkinen K (2012). Associations of maximal strength and muscular endurance test scores with cardiorespiratory fitness and body composition. J Strength Cond Res.

[56] White R, Cotton SM, Hind J, Robbins J, Perry A (2009). A comparison of the reliability and stability of oro-lingual swallowing pressures in patients with head and neck cancer and healthy adults. Dysphagia.

[57] Wirth M, Unterhuber D, Meyer F, von Hofauer 
B, Ott A, Edenharter G, Eckert DJ, Heiser C (2020). Hypoglossal nerve stimulation therapy does not alter tongue protrusion strength and fatigability in obstructive sleep apnea. J Clin Sleep Med.

[58] Woodson BT, Steward DL, Mickelson S, Huntley T, Goldberg A (2010). Multicenter study of a novel adjustable tongue-advancement device for obstructive sleep apnea. Otolaryngol Head Neck Surg.

[59] Woodson BT, Wooten MR (1992). A multisensor solid-state pressure manometer to identify the level of collapse in obstructive sleep apnea. Otolaryngol Head Neck Surg.

[60] Yoshida M, Kikutani T, Tsuga K, Utanohara Y, Hayashi R, Akagawa Y (2006). Decreased tongue pressure reflects symptom of dysphagia. Dysphagia.

[61] Youmans SR, Stierwalt JA (2006). Measures of tongue function related to normal swallowing. Dysphagia.

[62] Youmans SR, Youmans GL, Stierwalt JA (2009). Differences in tongue strength across age and gender: is there a diminished strength reserve?. Dysphagia.

[63] Young T, Peppard PE, Gottlieb DJ (2002). Epidemiology of obstructive sleep apnea: a population health perspective. Am J Respir Crit Care Med.

[64] Zhou J, Liu Y (2013). Effects of genistein and estrogen on the genioglossus in rats exposed to chronic intermittent hypoxia may be HIF-1$$\alpha $$ dependent. Oral Dis.

